# Evaluation of Weissella Cibaria JW15 Probiotic Derived from Fermented Korean Vegetable Product Supplementation in Diet on Performance Characteristics in Adult Beagle Dog

**DOI:** 10.3390/ani9080581

**Published:** 2019-08-20

**Authors:** Hao Yang Sun, Kun Phil Kim, Chun Ho Bae, Ae Jin Choi, Hyun Dong Paik, In Ho Kim

**Affiliations:** 1 Department of Animal Resource and Science, Dankook University, Cheonan 31116, Korea; 2 Aram Co., Ltd. 54 Gyeongchung-daero 1234 beon-gil, Chowol-eup, Gwangju-si, Gyeonggi-do 12735, Korea; 3 National Institute of Agricultural Science, Department of Agro-food Resources, 166 Nongsaengmyeong-ro, Iseo-myeon, Wanju-gun, Jeollabuk-do 55365, Korea; 4 Department of Food Science and Biotechnology of Animal Resources, Konkuk University, 120 Neungdong-ro, Gwangjin-gu, Seoul 05029, Korea

**Keywords:** adult Beagle dogs, probiotic, *Weissella cibaria* JW15, blood profiles, gut health

## Abstract

**Simple Summary:**

Dogs are the most popular companion animals worldwide, and their popularity is still increasing. Maintaining health and seeking optimal nutritional feed for dogs is an important component of responsible pet ownership. Probiotics have been widely used in animals; however, little research exists on some probiotic species which have been reported to have good probiotic properties. The present study investigated the effects of *Weissella cibaria* JW 15 isolated from the traditional Korean fermented vegetable product (kimchi) as a probiotic feed additive on the nutrition and health characteristics in the adult Beagle dogs. The results of this study indicated that *Weissella cibaria* probiotics have beneficial effects in dogs, which provide evidence and insight for the application of probiotics in dogs.

**Abstract:**

This study was conducted to evaluate the effects of dietary supplementation of *Weissella cibaria* JW15 (WJW15) isolated from traditional Korean fermented vegetable product (kimchi) as a probiotic feed additive on nutrient digestibility, blood profiles, feces noxious gas emission, and feces *Escherichia coli* and *Lactobacillus* counts in adult Beagle dogs. In total, 15 Beagle dogs with an average initial body weight of 10.20 ± 0.38 kg were randomly assigned into three dietary treatments in a 14-day feeding trial. Dietary treatments consisted of basal diet (CON); MJW = CON + 50 g of WJW15 (3.0 × 10^8^ cfu/g); and BJW = CON + 50 g WJW15 (3.0 × 10^9^ cfu/g). At the end of the experiment, the serum concentration of triglycerides and feces ammonia emissions were decreased (*P* < 0.05) with the increasing level of WJW15 supplementation. High-density lipoprotein cholesterol in serum and feces lactic acid bacteria count was improved (*P* < 0.05) with increasing levels of WJW15. In conclusion, WJW15 isolated from kimchi supplementation in adult Beagle dog diet may have beneficial effects as a probiotic feed additive.

## 1. Introduction

Kimchi is well-known as a traditional Korean fermented vegetable product, which is considered as one of the five healthiest foods from all over the world [1]. It was perceived that practically all kinds of kimchi contain high levels of vitamins, minerals, dietary fibers, and phytochemicals typically from garlic, ginger, and red pepper that have beneficial effects to human health [2]. Moreover, the lactic acid bacteria (LAB) that is involved during fermentation was also considered to have health-promoting properties [3]. The LAB species involved in traditional kimchi fermentation are generally considered safe for human consumption and mainly dominated by strains of the genera *Lactobacillus*, *Leuconostoc*, *Weissell,a* and *Pediococcus* [4].

Additionally, these LAB species are also considered as probiotic microorganisms [5]. Probiotics are defined as “‘living microorganisms’, which upon ingestion in certain numbers, exert health benefits beyond inherent basic nutrition” [6]. Moreover, many previous studies suggested that dietary supplementation of single or multi-LAB strains as probiotics, directly and indirectly, led to beneficial impacts in monogastric animals [7,8,9,10]. The genus *Weissella*, a member of the LAB family, consists of 19 species. Among these species, *Weissella cibaria* (*W. cibaria*) was first classified in 2002 and has been isolated from kimchi as a dominant species [11]. It was reported that *W. cibaria* JW15 (WJW15) exhibited probiotic properties and immune-modulatory effects such as antimicrobial activities and increasing nuclear factor (NF)-κB, Interleukin-1-beta, and tumor necrosis factor-α in macrophage cells [12,13]. Dogs are the most popular companion animals worldwide, and their popularity is still increasing [14]. Maintaining health and seeking optimal nutritional feed for dogs are important components of responsible pet ownership [15]. Previous studies have reported many strains of LAB used in dogs [16,17,18]. However, few studies reported the effects of JW15 as a probiotic feed additive for dogs. Thus, the objective of the present study was to investigate the effects of two levels of WJW15 isolated from Korean kimchi on nutrient digestibility, blood profiles, feces lactic acid bacteria and coliform bacteria counts, and feces noxious gas emissions in healthy adult Beagle dogs.

## 2. Materials and Methods

The experimental protocol (DK-1-1824) describing the management and care of animals was reviewed and approved by the Animal Care and Use Committee of Dankook University, Cheonan, South Korea.

### 2.1. Experimental Design, Animals, Diets, and Housing

In total, 15 Beagle dogs (Orient Bio, Seoul, Korea) at one-to two-year-old with initial body weight (BW) of 10.20 ± 0.38 kg were used in a 14-day feeding trial. Based on BW and sex, the dogs were randomly assigned into three dietary treatments: CON, commercial basal diet; MJW, CON + 50 g of WJW15 with 3.0 × 10^8^ cfu/g; and BJW, CON + 50 g WJW15 with 3.0 × 10^9^ cfu/g. Each treatment consisted of five replication and one dog per cage (three males and two females). The WJW15 were presented in powder form and mixed properly with feed by using a mixer. A commercial basal diet was formulated in accordance with the Association of American Feed Control Officials (AAFCO, 2009) nutrient guide for dogs and balance to meet maintenance requirements. The nutrient composition of the basal diet is shown in Table 1. Dogs were individually fed twice a day (8 am and 4 pm) in sufficient amounts to supply their metabolic energy (ME) requirements, according to the National Research Council (NRC, 2006). Beagles were housed in cages (100 cm × 210 cm) that were equipped with a feeder, a water bucket, and slatted plastic flooring in an environmentally controlled room. Dogs were allowed ad libitum access to drinking water throughout the experiment. Room temperature and relative humidity were maintained at 20 ± 3 °C and 50 ± 10%, respectively.

### 2.2. Source of Probiotics

*Weissella cibaria* KACC 91811P (JW15) was obtained from the Korean Agricultural Culture Collection (KACC; Jeollabuk-do, Korea) in a freeze-dried ampoule. It was cultivated and manufactured by a commercial company (Lacto Mason Co., Ltd. Kyungnam, Jinju, Korea).

### 2.3. Sampling and Measurements

The nutrient digestibility was performed using the total collection method (AAFCO, 2009). To calculate the nutrient digestibility of dry matter (DM) and nitrogen (N), during the last three days of the experiment, feces were collected at least twice daily, weighed and stored in a freezer at −20 °C. Feces samples from the same dog were pooled and mixed, after which samples were stored at −20 °C until analysis. For chemical analysis, feces samples were thawed and dried at 70 °C for 72 h, ground to a fine powder, and passed through a 1-mm screen. All feed and feces samples were analyzed for DM (method 930.15) and N (method 984.13) using the AOAC (2007) method. The digestibility of DM and N was calculated as follows: 

Digestibility of X (%) = [(X intake, g − X excretion, g)/X intake, g] × 100

At the end of the experiment (day 14), feces samples for microbial and noxious gas emission were collected from each dog. For feces microbial, feces was placed in a micro-tube, on ice for transportation to the laboratory where the analysis was immediately carried out. One gram of feces was diluted with 9 mL of 1% peptone broth (Becton, Dickinson and Co., Franklin, Lakes, NJ, USA) and homogenized. Counts of viable bacteria in feces samples were determined by plating 10-fold serial dilutions (in 1% peptone broth solution) onto MacConkey agar plates (Difco Laboratories, Detroit, MI, USA) and Lactobacilli medium agar plates (Medium 638; DSMZ, Braunschweig, Germany) to isolate coliform bacteria and lactic acid bacteria, respectively. The Lactobacilli medium agar plates were incubated for 48 h at 39 °C under anaerobic conditions. The MacConkey agar plates were incubated for 24 h at 37 °C. Lactic acid bacteria and coliform bacteria colonies were counted immediately after removal from the incubator. For feces noxious gas emission, feces samples were collected from each pen. Samples were stored in 2.6 L plastic boxes, in duplicates. Each box had a small hole in the middle of one sidewall, which was sealed with adhesive plaster. The samples were permitted to ferment for a period of 5 days at room temperature, 25 °C. After the fermentation period, a GV- 100 gas sampling pump (Gastec Corp., Kanagawa, Japan) was used for the detection of ammonia (NH_3_), hydrogen sulfide (H_2_S), and total mercaptans (R.SH) using different detection tubes (No. 3L, No. 4LT, and No. 70L; Gastec). To this end, the seal was punctured, and 100 mL of headspace air was sampled approximately 2 cm above the feces. After air sampling, each box was sealed again, covered with adhesive tape. Headspace measurements were repeated after 58 h. The gas contents were averaged from two measurements.

At the end of the experiment (day 14), blood samples (5 mL) were taken from each dog by vacuum tubes and K_3_EDTA (salt of ethylenediaminetetraacetic acid tripotassium) tubes (Becton Dickinson Vacutainer Systems, Franklin Lakes, NJ, USA) to obtain serum and whole blood, respectively. White blood cells (WBC), red blood cells (RBC), and lymphocyte counts of whole blood samples were determined using an automatic blood analyzer (ADVIA 120, Bayer, Tarrytown, NY, USA). Serum was separated by centrifugation for 30 min at 2000 g at 4 °C and stored until further analysis. Blood lipid profiles, including total cholesterol (TC), triglyceride (TG), high-density lipoprotein cholesterol (HDL-C), and low-density lipoprotein cholesterol (LDL-C) were analyzed by using an automatic biochemistry analyzer (Hitachi 747; Hitachi Ltd., Tokyo, Japan) with commercial kits (MAK043, MAK045, and TR0100, Sigma Diagnostics, MO, USA) according to the manufacturer’s protocol.

### 2.4. Statistical Analysis

Data were analyzed as a randomized complete block design using the general linear model (SAS Institute Inc., Cary, NC, USA). For nutrient digestibility, blood profiles, and the samples of feces noxious gas emission from individual dogs was used as the experimental unit. For microbial counts, data were log-transformed prior to statistical analysis. Linear and quadratic polynomial contrasts were performed to determine the effect of supplementation level (0, 3.0 × 10^8^ cfu/g, and 3.0 × 10^9^ cfu/g) of WJW15. Variability in the data was expressed as a pool as means ± standard deviation (SD) and a probability level of less than 0.05 was considered statistically significant.

## 3. Results

No significant difference (*P* > 0.05) was observed on nutrient digestibility of DM and N following the increasing levels of WJW15 supplementation in dogs (Table 2). At the end of the experiment, the concentration of serum TG was linearly decreased (*P* = 0.0314) with an increase in the levels of WJW15 supplementation; on the contrary, following the increasing levels of JWJ15, the serum HDL-C concentration was linearly increased (*P* = 0.0037). Other blood profiles such as TC, LDL-C, WBC, RBC, and Lymphocyte were not affected (*P* > 0.05) by the supplementation of JWJ15 (Table 3). As described in Table 4, different levels of dietary WJW15 did not influence (*P* > 0.05) the population of feces coliform bacteria; whereas, following the increasing level of WJW15 supplementation, feces lactic acid bacteria count was linearly increased (*P* = 0.0006). The data presented in Table 5 show that the feces noxious gas emission of H_2_S and R.SH was not affected (*P* > 0.05) by the WJW15 supplementation; however, the concentration of feces NH_3_ emission linearly decreased (*P* = 0.0302) with the increasing level of dietary WJW15.

## 4. Discussion

The digestibility of nutrients based on WJW15 supplementation in dogs has been not researched; therefore, direct comparisons couldn’t be made. Previously, in agreement with this study, Biourge et al. reported that supplementation of 7.5 × 10^8^ cfu per day of probiotics did not influence the digestibility of DM and protein, as well as lipid and energy in dogs [19]. However, in a pig experiment, Giang et al. observed that dietary inclusion of LAB probiotics improved the apparent total tract digestibility of protein, organic matter, and fiber [9]. It was reported that in farm animals (swine and poultry), one of the mechanisms of action of probiotics is to improve digestion and utilization of nutrients, which consequently improves the growth rate and product quality [20]. Whereas, for adult dogs, it was considered that the primary mission was to have better health and to help reduce the risk of diseases. Thus, it could be suggested that the unaffected results on the nutrient digestibility of WJW15 supplementation in this experiment may not affect the consumer acceptability. The inconsistent finding could be due to the feed types, species, and age of the animals.

Blood profiles such as serum lipid concentrations, WBCs, and lymphocytes could reflect the physiological state and immunologic system status of dogs. WBCs are immune cells that protect the body against infectious disease and foreign invaders; furthermore, the serum lipid concentration has been linked with the health status of humans and animals [21]. It was reported that dietary supplementation of probiotics may have an interactive action between probiotic-immune systems [22]. But the blood profiles of dogs based on WJW15 supplementation has not been reported before. El-Gawad et al. reported that rats fed LAB probiotic-supplemented diet had significantly lower plasma and liver cholesterol levels than those without LAB supplementation [23]. However, in a laying hen experiment, Zhang et al. reported that blood lipids of HDL-C, LDL-C, and triglyceride were not affected by LAB probiotics which is not consistent with the result of this experiment on triglycerides [24]. In the current study, the mean values for triglycerides in the WJW15-fed dogs were lower than those of the CON-fed diet. The mechanisms involved in blood lipid levels have not been fully elucidated. One possible reason may be because the probiotic bacteria could be fermentation indigestible carbohydrates, produce a short-chain fatty acid, and redistribution cholesterol from plasma to liver, which could decrease the levels of blood lipids [5]. However, the results of this study failed to obtain any differences in RBC, WBC, and lymphocyte in WJW15 added to the diet of dogs. Previous studies reported that LAB probiotics had beneficial impacts on swine and poultry [25,26]. It was suggested that probiotics influence the immune system by metabolites, cell wall components, or DNA, which may reduce the risk of disease [27]. The lack of consistency among species and studies may be due to the diet, age, and species of animals.

It is generally suggested that the gastrointestinal tract and the overall health of the animals were strongly linked [15]. The gut microbiome plays important roles in the host, not only through nutrient absorption but also throiugh downstream effects of microbial metabolite generations [28]. Lactic acid bacteria are considered as beneficial bacteria, whereas coliform bacteria are known to be harmful to animals [29,30]. It has been proved that LAB probiotics help to accelerate the development of the normal microbiome in monogastric animals through the production of antimicrobial substances such as organic acids, nitrogen oxide, and nisin [31,32,33]. In agreement with this study, Biagi et al. reported that LAB probiotic influenced the composition and metabolism of the intestinal microbiome of adult dogs [34]. Recently, Yu et al. suggested that WJW15 as a probiotic possessed the potential of amelioration of disorders induced by pathogenic bacteria [35]. The increased count of feces lactic acid bacteria in this experiment may be due to the supplementation of WJW15. However, the mode of action of LAB probiotics is not always understood due to different strains having various functions and survivability throughout the gut affecting the host in different ways [36]. The results of this experiment suggested that the addition of WJW15 might have a beneficial effect on dog gut health, which improves the health condition and well-being of Beagle dogs. Further experiments are required to explain the exact mechanism by which WJW15 supplementation affects the gut microbiome of dogs.

The noxious gas emissions such as NH_3_ and H_2_S in animal husbandry is an environmental concern since it is relevant to the soil and water acidification and increases N and S deposition in ecosystems. Yan et al. considered that animal feces noxious gas emission is related to the nutrient digestibility [37]. It was suggested that the improved digestibility of nutrients might result in less substrate for microbial fermentation, which consequently decreases the feces noxious gas emission. However, the results of nutrient digestibility in this study failed to support this point. On the other hand, it also reported that noxious gas emission is related to a harmful intestinal bacteria population, which corroborates with the findings of the present study [38]. NH_3_ is produced as a by-product of the microbial decomposition of nutrient compounds in feces and urine [39]. The possible explanation for the decreased NH_3_ emission in this study may be due to the improved feces lactic acid bacteria count in dogs.

## 5. Conclusions

In conclusion, Beagle dog diets supplementation with WJW15 isolated form Korean kimchi decreased the serum TG concentration, increased the HDL-C concentration, increased the feces lactic acid bacteria counts, and decreased the concentration of feces NH_3_ emission. It is suggested that the supplementation of WJW15 in Beagle dog diets could enhance the blood lipid parameters which may positively affect the health status of adult dogs.

## Figures and Tables

**Table 1:** Basal diet composition (as-fed basis).

Raw Material, %	Content
Corn	3.00
Wheat	15.727
Rice	8.85
Wheat bran	6.00
Beet pulp	3.63
Soybean meal (45% of crude protein)	10.09
Meat bone meal	7.00
Meat meal (60%, low protein)	3.40
Meat meal (70%, high ash)	6.00
Poultry meal	20.00
Tallow	9.20
Poultry fat	5.50
Salt	0.50
Methionine (99%, L-Form)	0.09
Mineral-Vitamin premix ^1^	0.20
Vitamin E (10%)	0.093
Enzyme	0.03
Dried beer yeast	0.50
Herb	0.12
Yucca	0.02
Antioxidant	0.05
Total	100.00
Calculated composition, %	
Dry matter	90.59
Crude protein	32.01
Crude fat	19.97
Crude fiber	2.20
Crude ash	8.79
Calcium	1.96
Total phosphorus	1.26

^1^ Formulated to supply a minimum of 0.5 g of magnesium, 1.2 g of sodium, 8.0 g of potassium, 2.3 g of chloride, 165 mg of iron, 141 mg of zinc, 7.7 mg of copper, 13 mg of manganese, 0.2 mg of selenium, 1.5 mg of iodine, 0.2 mg of biotin, 1226 mg of choline, 1.7 mg of folic acid, 45 mg of niacin, 15 mg of pantothenic acid, 7.8 mg of pyridoxine, 6.0 mg of riboflavin, 38 mg of thiamin, and 0.09 mg of vitamin B_12_/kg of diet and to supply 16.4 U of vitamin A, 1.0 U of vitamin D, and 0.18 U of vitamin E/g of diet.

**Table 2:** Effects of dietary supplementation of *Weissella cibaria* JW15 probiotic on nutrient digestibility in dogs ^1^.

Items, %	CON	MJW	BJW	*P*-value	
				Linear	Quadratic
Dry matter	86.99 ± 0.77	87.12 ± 0.70	87.31 ± 0.64	0.4080	0.9337
Nitrogen	85.51 ± 0.57	85.56 ± 0.38	85.62 ± 0.36	0.6180	0.9777

^1^ Abbreviation: CON, Basal Diet; MJW, CON + 50 g of WJW15 with 3.0 × 10^8^ cfu/g; BJW, CON + 50 g of WJW15 with 3.0 × 10^9^ cfu/g.

**Table 3:** Effects of dietary supplementation of *Weissella cibaria* JW15 probiotic on blood profiles in dogs ^1^.

Items	CON	MJW	BJW	*P*-value	
				Linear	Quadratic
Cholesterol, mg/dL	235.75 ± 10.78	219.00 ± 11.36	215.75 ± 20.58	0.2060	0.6005
Triglyceride, mg/dL	57.25 ± 5.80	45.00 ± 9.62	43.25 ± 5.54	0.0314	0.2718
HDL-C^2^, mg/dL	131.75 ± 4.44	136.75 ± 5.49	146.00 ± 3.67	0.0037	0.4597
LDL-C^2^, mg/dL	5.75 ± 0.43	5.75 ± 1.30	5.25 ± 0.43	0.3794	0.6036
WBC^2^, 10^3^/μL	12.16 ± 0.37	14.00 ± 3.61	16.27 ± 0.37	0.1641	0.9260
RBC^2^, 10^6^/μL	6.77 ± 0.22	6.72 ± 0.27	6.69 ± 0.37	0.6810	0.9286
Lymphocyte^3^, %	57.48 ± 4.31	59.60 ± 6.79	63.30 ± 10.89	0.3695	0.8846

^1^ Abbreviation: CON, Basal Diet; MJW, CON + 50 g of WJW15 with 3.0 × 108 cfu/g; BJW, CON + 50 g of WJW15 with 3.0 × 109 cfu/g. ^2^ HDL-C, high-density lipoprotein cholesterol; LDL-C, low-density lipoprotein cholesterol; RBC, red blood cell; WBC, white blood cell. ^3^ Values are presented as a percentage of the total WBC count.

**Table 4:** Effects of dietary supplementation of *Weissella cibaria* JW15 probiotic on feces microbial in dogs ^1^.

Items, log_10_cfu/g	CON	MJW	BJW	*P*-value	
				Linear	Quadratic
Lactic acid bacteria	8.79 ± 0.12	8.93 ± 0.13	8.99 ± 0.09	0.0006	0.4018
Coliform bacteria	7.86 ± 0.15	7.83 ± 0.11	7.80 ± 0.12	0.3362	0.9845

^1^ Abbreviation: CON, Basal Diet; MJW, CON + 50 g of WJW15 with 3.0 × 10^8^ cfu/g; BJW, CON + 50 g of WJW15 with 3.0 × 10^9^ cfu/g.

**Table 5:** Effects of dietary supplementation of *Weissella cibaria* JW15 probiotic on gas emissions in dogs ^1^.

Items, ppm	CON	MJW	BJW	*P*-value	
				Linear	Quadratic
Ammonia	26.0 ± 4.0	16.5 ± 2.5	16.0 ± 5.0	0.0302	0.1000
Hydrogen sulfide	1.15 ± 0.15	0.85 ± 0.05	0.35 ± 0.25	0.1659	0.7868
Total mercaptan	4.80 ± 0.20	4.75 ± 0.05	3.45 ± 1.05	0.2858	0.5211

^1^ Abbreviation: CON, Basal Diet; MJW, CON + 50 g of WJW15 with 3.0 × 10^8^ cfu/g; BJW, CON + 50 g of WJW15 with 3.0 × 10^9^ cfu/g.
